# Rapid removal of Pb(II) from aqueous solution using branched polyethylenimine enhanced magnetic carboxymethyl chitosan optimized with response surface methodology

**DOI:** 10.1038/s41598-017-09700-5

**Published:** 2017-08-31

**Authors:** Yaoguang Wang, Di Wu, Qin Wei, Dong Wei, Tao Yan, Liangguo Yan, Lihua Hu, Bin Du

**Affiliations:** 1 Key Laboratory of Interfacial Reaction & Sensing Analysis in Universities of Shandong, School of Chemistry and Chemical Engineering, University of Jinan, Jinan, 250022 China; 2 School of Resources and Environment, University of Jinan, Jinan, 250022 China

## Abstract

In this study, branched polyethylenimine (PEI) enhanced magnetic carboxymethyl chitosan (MCMC-PEI) was synthesized and applied as adsorbent for the rapid removal of Pb(II) from aqueous solution. The successful synthesis of the adsorbent was proved by scanning electron microscope (SEM), Fourier transform infrared spectrum (FTIR) and X-ray powder diffraction (XRD). Simultaneously, the effect of the parameters such as initial concentration, adsorbent mass and pH of the solution on the removal of Pb(II) was studied by using response surface methodology (RSM). And central composite design (CCD), which is a widely used form of RSM, was employed in the experimental design procedure. The adsorption results revealed that the adsorption process could reach equilibrium rapidly within 10 min. Furthermore, the adsorption kinetic data could be well described by pseudo-second order model. The maximum adsorption capacity was 124.0 mg/g according to the Langmuir-Freundlich model, which fitted the adsorption isotherm of Pb(II) better than Langmuir model and Freundlich model, respectively. Thermodynamic studies (Δ*G* < 0, Δ*H* < 0, Δ*S* > 0) implied a spontaneous and exothermic process in nature. Meanwhile, the fabricated adsorbent exhibited excellent reusability. Therefore, the excellent adsorption property of MCMC-PEI made it a promising application in water treatment.

## Introduction

Heavy metal pollution due to the indiscriminate disposal of wastewater is a worldwide environment concern. Wastewater from industries such as metallurgical, mining, chemical manufacturing and battery manufacturing contains many kinds of toxic heavy metal ions^1–3^. Water pollution caused by heavy metal ions such as Pb(II) has become a crucial worldwide environmental problem with significant effect on human health and environment^4–6^. For abatement of heavy metal contaminants from aqueous solution, the techniques of precipitation, membrane filtration, adsorption, and ion exchange were generally used^7, 8^. Hereinto, adsorption has been recognized as a promising and cost-effective technique for the treatment of wastewater with heavy metal contaminants^9, 10^.

Conventional adsorbents have a major drawback that it is difficult to separate adsorbents from the liquid. The magnetic adsorbents have been widely used to solve the water pollution due to the simple solid-liquid phase separation^11–14^. However, the adsorption capacity of single magnetic material is usually poor, hence a large amount of magnetic composite adsorbents has been investigated to improve the adsorption capacity^15–18^. Chitosan has been reported for the high potentials of adsorbing metal ions, principally due to the presence of hydroxyl and amine groups which can serve as chelating sites^19–21^. However, the hydrophilic property of chitosan is not very good for practical application. In order to improve the hydrophilic property and further enhance the adsorption capacity for metal ions, chemical modification of chitosan is required. Carboxymethyl chitosan (CMC) is an amphiprotic chitosan derivative, which contains hydroxyl (–OH), carboxyl (–COOH) and amine (–NH_2_) groups in the molecule. These functional groups can improve the hydrophilic property of CMC and provide enough adsorption sites for increasing adsorption capacity^22^. Polyethylenimine (PEI) which is composed of plenty of amine groups on the macromolecular chains has been reported to have strong adsorption ability for heavy metals^23, 24^. However, because of the water soluble nature of PEI, it has to be immobilized on matrix to ensure the maneuverability when used as adsorbent^25^. For example, insoluble polymers^26^, biomass^27^ and cellulose^28^ have been used to crosslink PEI to prevent its leaching during adsorption operation.

In addition, it is well-known that adsorption efficiency depends on various experimental factors, such as adsorbent dosage, initial adsorbate concentration, temperature, and solution pH value^29, 30^. Conventional adsorption experiments were usually carried out by varying one experimental factor and keeping the other constants to determine the influence of each one of the factors^31, 32^. The obvious shortcomings associated with these conventional methods are the unreliability of the results, nondepiction of the combined effect of the independent variables, and greater time consumption due to more experiments^33–35^. Experimental design can help the researchers to estimate the main and interaction of variables for the simultaneous optimization and investigation of variables effect with at least experiments. Response surface methodology (RSM) is an empirical statistical technique used to evaluate the relationship between a set of controlled experimental factors and observed results^36^.

**Figure 1:**
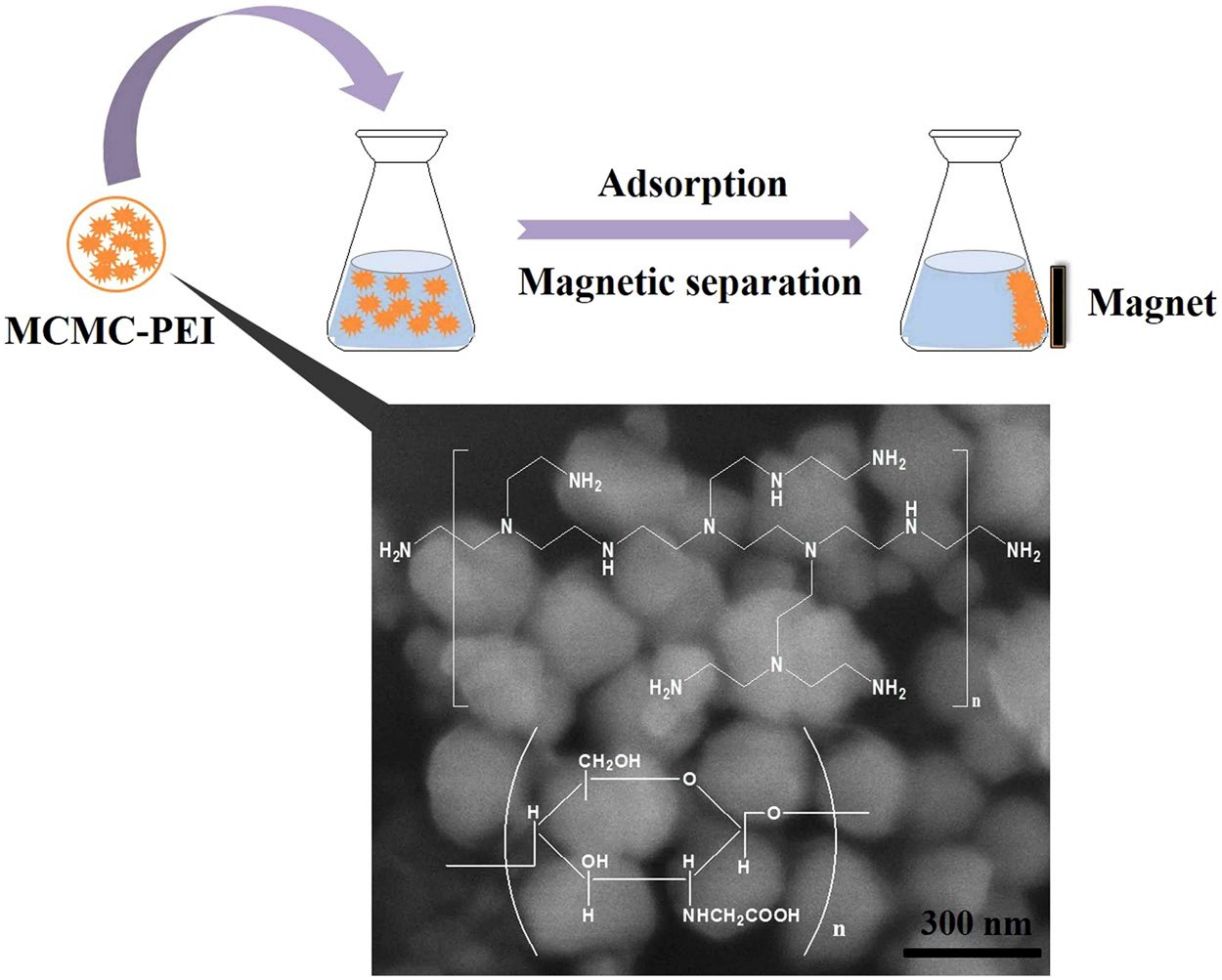
Schematic representation of the branched PEI enhanced magnetic carboxymethyl chitosan.for rapid removal of Pb(II) from aqueous solution.

In this study, branched PEI enhanced magnetic carboxymethyl chitosan (MCMC-PEI) was synthesized and applied for the rapid removal of Pb(II) from aqueous solution (Fig. 1). The crosslinked magnetic carboxymethyl chitosan (MCMC) was prepared firstly. Then branched PEI was grafted on MCMC to obtain MCMC-PEI. The influence of important variables including pH, adsorbent dosage and initial Pb(II) concentration were investigated and optimized by central composite design (CCD) combined with RSM according to the desirability function as maximize criterion of the response.

## Experimental

### Materials

Nanosized ferroferric oxide was purchased from Aladdin Industrial Inc. and branched polyethylenimine (M.W. 10000, 99%) was obtained from Macklin Inc. Carboxymethyl chitosan (CMC), which substitution degree was not less than eighty percent, was obtained from Nantong Lvshen Biological Engineering Co., Ltd. 1-ethyl-3-[3-dimethylaminopropyl] carbodiimide hydrochloride (EDC) and N-hydroxyl succinimide (NHS) were purchased from Shanghai Civi Chemical Technology Co., Ltd. Other chemicals were obtained from Sinopharm Chemical Reagent Beijing Co. Ltd., China and were of analytical reagent grade. Ultrapure water (EASY-pure LF, Barnstead International, Dubuque, IA, USA) was used throughout the experiment.

### Preparation of MCMC

The preparation process of MCMC was as follows: Firstly, 0.5 g of CMC was dissolved in 50 mL of ultrapure water, and the mixture was sonicated at room temperature for 3 h. Then, 0.2 g of magnetic nanosized ferroferric oxide was added to the colloidal solution and the reaction system was continually stirred using electric blender for 1.5 h. Subsequently, 3 mL of liquid paraffin was dispersed slowly in the mixture under stirring. After 0.5 h of emulsification, 3 mL of glutaraldehyde as cross-linker was added dropwise. Afterwards, the mixed system was stirred continuously for 2 h in an oil bath at 60 °C. The product was washed with petroleum ether, ethanol and ultrapure water in turn. Eventually, the precipitate was dried in vacuum and the product (MCMC) was ground to a fine powder.

### Preparation of MCMC-PEI

The synthetic procedures of MCMC-PEI were described as follows: 0.3 g of MCMC was dispersed in 20 mL of ultrapure water and stirred with an electric blender. Then 0.2 g of EDC and 0.3 g of NHS were added into the dispersion. Subsequently, the mixture was adjusted to pH 5–6 and stirred continuously in order to activate the carboxyl groups of MCMC. After 1 h, 1.0 g of PEI was added and the pH of the system was adjusted to 7–8, and the reaction lasted another 12 h. The final product was washed with ultrapure water and ethanol, and dried in vacuum.

### Characterization methods

The morphology observation of MCMC-PEI was carried out by using a QUANTA FEG 250 scanning electron microscopy (FEI, United States). Fourier transform infrared spectrum (FTIR) spectra of the samples were obtained from a Perkin-Elmer Spectrum One FTIR spectrometer (Perkin–Elmer, United States). The spectra were recorded from 4000 to 450 cm^−1^. X-ray powder diffraction (XRD) patterns were recorded on a D8 FOCUS X-ray diffraction spectrometer (Bruker, Germany) with Cu Kα radiation for crystalline phase identification. The sample was scanned from 10° to 80°.

### Batch adsorption experiments

Batch adsorption experiments for Pb(II) removal using MCMC-PEI were conducted using a thermostatic water bath oscillator. A predetermined amount of adsorbent was added to 25 mL solution of known Pb(II) concentration in 100 mL air-tight conical flasks. NaNO_3_ (0.1 mol/L) was added into the Pb(II) solutions as constant background electrolyte and the pH was adjusted using HNO_3_ and NaOH. The mixed system was agitated for certain time at different temperatures. After the contact time defined by experimental design, the adsorbent was separated by a magnet. The equilibrium concentrations of Pb(II) were determined using an atomic absorption spectrophotometer.

The removal efficiency and the adsorption amount *q*_t_ (mg/g) were calculated based on the difference of the Pb(II) concentration in the aqueous solution before and after adsorption according to the formula:

1 $${q}_{{\rm{t}}}=({c}_{{\rm{o}}}-{c}_{{\rm{t}}})\times V/m$$

Where *c*_o_ and *c*_t_ (mg/L) are the concentration of adsorbate at initial and time *t* (min), respectively. *V* (L) is the volume of adsorbate solution, *m* (g) is the mass of adsorbents, *q*_t_ (mg/g) is the adsorbed amount at time *t* (min).

### Experimental design

In order to study the effect of the parameters (initial concentration, adsorbent mass and pH of the solution) on the removal of Pb(II), experiments were carried out using RSM. Central composite design (CCD) which is widely used form of RSM was employed in the experimental design procedure. The total number and sequence of experimental runs were determined using Design Expert 8.0.6 software (trial version, Stat-Ease Inc, Minneapolis, MN, USA). Initial solution pH (*X*_1_), adsorbent dosage (*X*_2_), and initial Pb(II) concentration (*X*_3_) were selected as independent input variables. The amount of Pb(II) ions adsorption after 3 h at 303 K (*Y*) were taken as dependent output response variables of the system. The experimental ranges and the levels of the independent variables for Pb(II) ion removal on MCMC-PEI are given in Table 1. A total of 20 experiments were employed in the study, including 2^3^ = 8 cube points, 6 replications at the center point and 2 × 3 = 6 axial points.

**Table 1:** Experimental range and levels of the independent variables.

Factors range and levels (coded)	*X*_1_, pH	*X*_2_, Adsorbent dosage (g/L)	*X*_3_, Pb(II) concentration (mg/L)
−1.682	3	0.2	30
−1	3.61	0.28	38.11
0	4.5	0.4	50
1	5.39	0.52	61.89
1.682	6	0.6	70

In a system involving three independent variables, the mathematical relationship of the response *Y* to these variables can be approximated by the quadratic polynomial equation:

2 $$Y={b}_{0}+\sum _{i}^{k}{b}_{i}{x}_{i}+\sum _{i}^{k}{b}_{ii}{x}_{i}^{2}+\sum _{i}\sum _{j}{b}_{ij}{x}_{i}{x}_{j}+{\varepsilon }_{r}$$

where *Y* is a response variable of adsorption capacity; *i* and *j* take value from 1 to the number of independent process variables; the *b*_*i*_ values are regression coefficients for linear effects; *b*_*ii*_ and *b*_*ij*_ values are the regression coefficients for quadratic effects; *x*_*i*_ and *x*_*j*_ are coded experimental levels of the variables; *ε*_r_ is the error of prediction.

Statistical analysis, including the analysis of variance (ANOVA), *t*-test, *F*-test and the determination of the coefficients (*R* ^2^), was performed using the software Design-Expert 8.0.6.

## Results and Discussion

### Characterization of adsorbent

**Figure 2:**
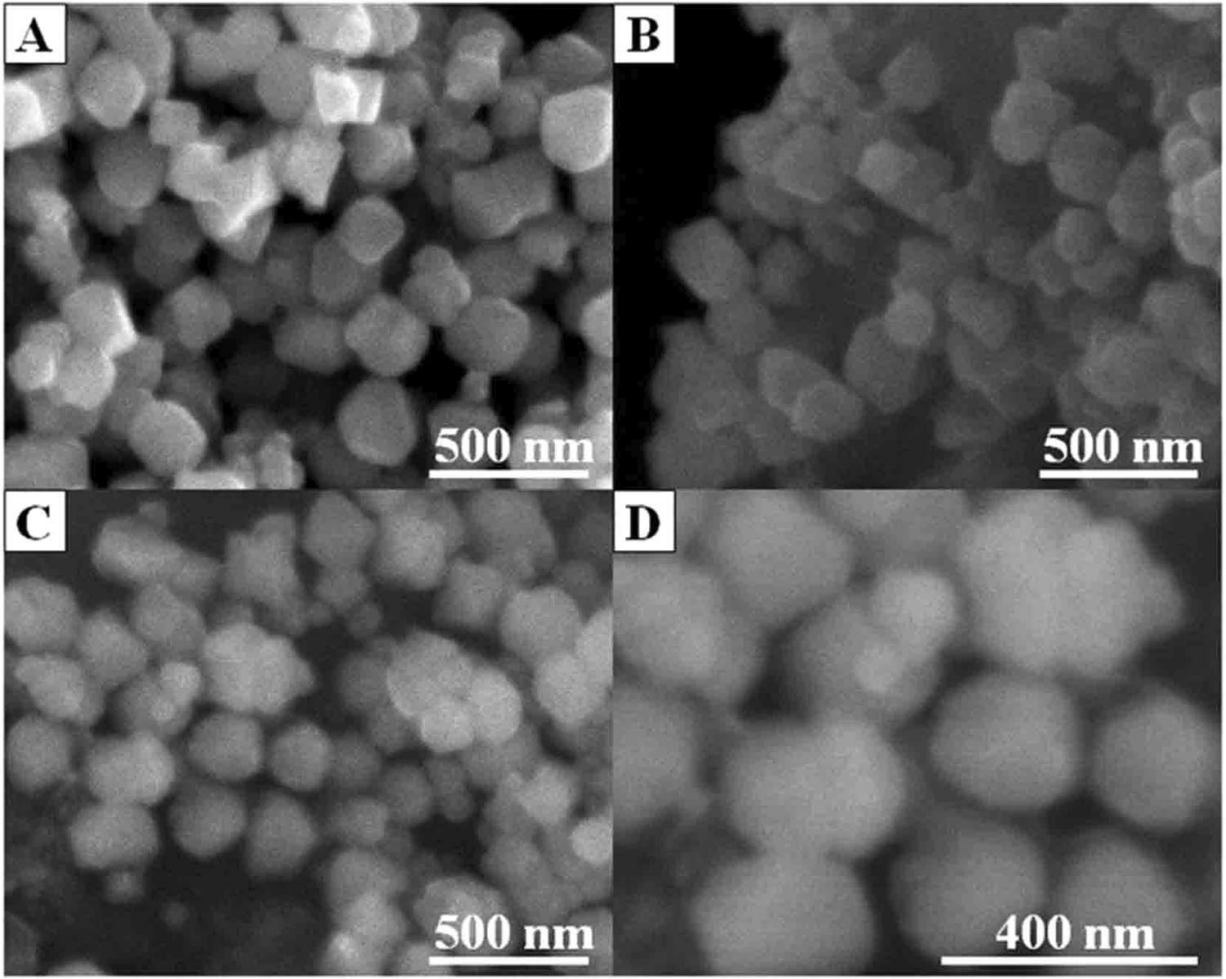
SEM images of Fe_3_O_4_ (**A**), MCMC (**B**) and MCMC-PEI (**C** and **D**).

The morphology of the adsorbent was investigated by scanning electron microscopy (SEM). Figure 2A showed the SEM image of Fe_3_O_4_ nanoparticle, which had a polyhedral structure. The shape of nanoparticle could be clearly observed. After the combination with CMC, the Fe_3_O_4_ NPs were wrapped together in CMC, and the three-dimensional structure of nanoparticle became blurred (as shown in Fig. 2B). The morphology of MCMC-PEI was displayed in Fig. 2C and D. After grafted with PEI, the polyhedral structure had disappeared and the shape of nanoparticle transformed into approximate sphere. Furthermore, the conductivity of CMC and PEI was poor, hence the SEM images of materials became blurred with gradually modified.

**Figure 3:**
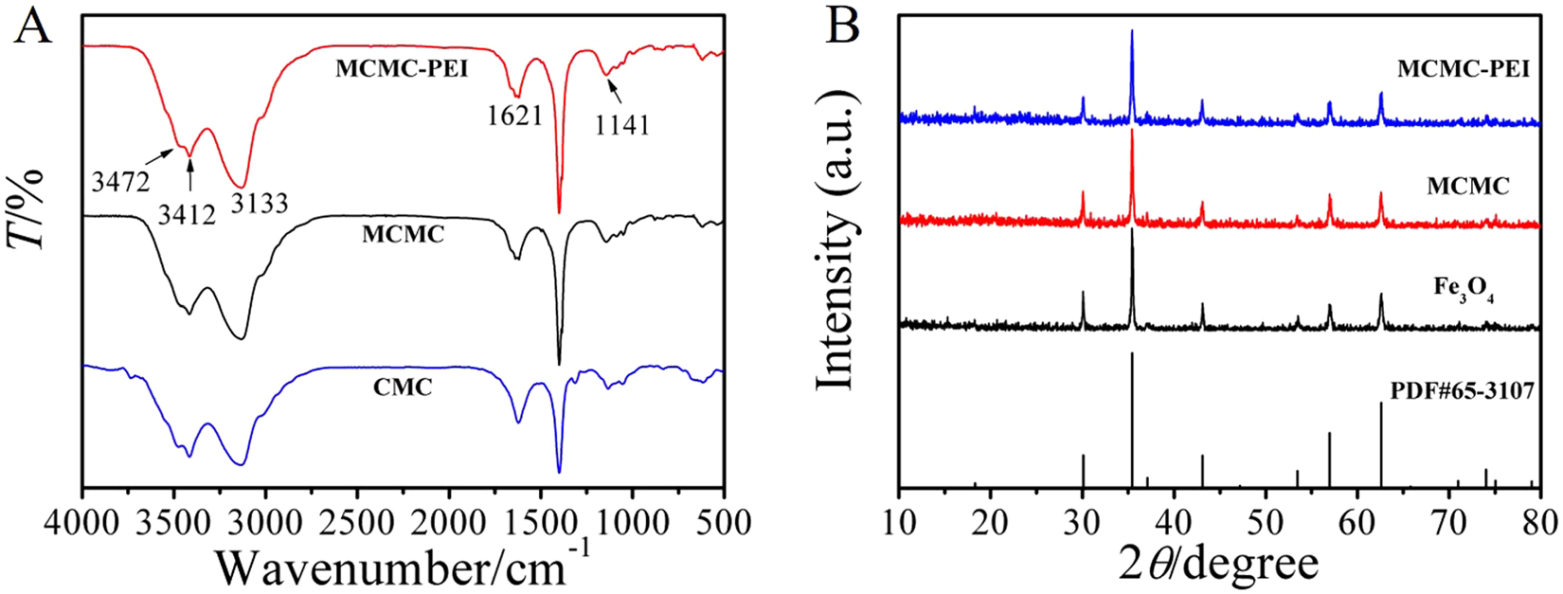
FTIR spectra (**A**) of CMC, MCMC and MCMC-PEI; XRD patterns (**B**) of Fe_3_O_4_, MCMC and MCMC-PEI.

Figure 3A displayed the FTIR spectra of CMC, MCMC and MCMC-PEI, revealing the presence of the important functional groups. The absorption band at 3472 cm^−1^ and 3412 cm^−1^ were attributed to the symmetrical and asymmetric stretching vibration of –NH_2_, demonstrating the existence of CMC and PEI. The peak at 3133 cm^−1^ corresponded to stretching vibration of O–H with the effect of hydrogen bonds, which made the absorption peak shift to lower wave numbers. The characteristic absorbance band at 1621 cm^−1^ was due to the stretching vibration of C = O in amide or carboxyl. Furthermore, the peak at 1141 cm^−1^ was attributed to the C–O stretching vibration. The above results further confirmed the successful preparation of MCMC-PEI.

XRD patterns of Fe_3_O_4_, MCMC and MCMC-PEI were shown in Fig. 3B. The positions and relative intensities of diffraction peaks matched well with those from the Jade PDF card for Fe_3_O_4_, indicating the existence of Fe_3_O_4_^37^. Meanwhile, the diffraction peaks of CMC and PEI did not appear in the corresponding spectrums of MCMC and MCMC-PEI, which was possibly because that CMC and PEI were amorphous in MCMC and MCMC-PEI^38^, respectively.

### CCD model and statistical analysis

The sequence of experiments and summary of the results were given in Table S1. Based on these results, empirical relationships between the responses and independent variables were obtained and expressed by the following second-order polynomial regression equations:

3 $$\begin{array}{rcl}Y & = & -\,\,290.4+136.4{x}_{1}+93.86{x}_{2}+0.1993{x}_{3}-48.80{x}_{1}{x}_{2}+0.3525{x}_{1}{x}_{3}\\  &  & +2.389{x}_{2}{x}_{3}-12.70{x}_{1}^{2}-35.37{x}_{2}^{2}-0.02326{x}_{3}^{2}\end{array}$$

The results of analysis of variance were shown in Table S2. As shown in the table, *x*_1_, *x*_2_, *x*_3_, *x*_1_ *x*_2_, *x*_1_ *x*_3_, *x*_2_ *x*_3_, *x*_1_^2^ and *x*_3_^2^ were significant parameters for the response of adsorption capacity. The term of *x*_2_^2^, whose values of *p* value were higher than 0.1000, were not significant. Eliminating the insignificant terms from the regression Eq. 3 and refining the model, the above empirical model equations may be simplified as shown:

4 $$\begin{array}{rcl}Y & = & -283.0+135.9{x}_{1}+65.56{x}_{2}+0.1642{x}_{3}-48.80{x}_{1}{x}_{2}+0.3525{x}_{1}{x}_{3}\\  &  & +2.389{x}_{2}{x}_{3}-12.63{x}_{1}^{2}-0.02291{x}_{3}^{2}\end{array}$$

The predicted values calculated with Eq. 4 were listed in the last column of Table S1 and the result of analysis of variance after amendment was shown in Table S3. It indicated good agreements between the experimental and predicted values.

**Figure 4:**
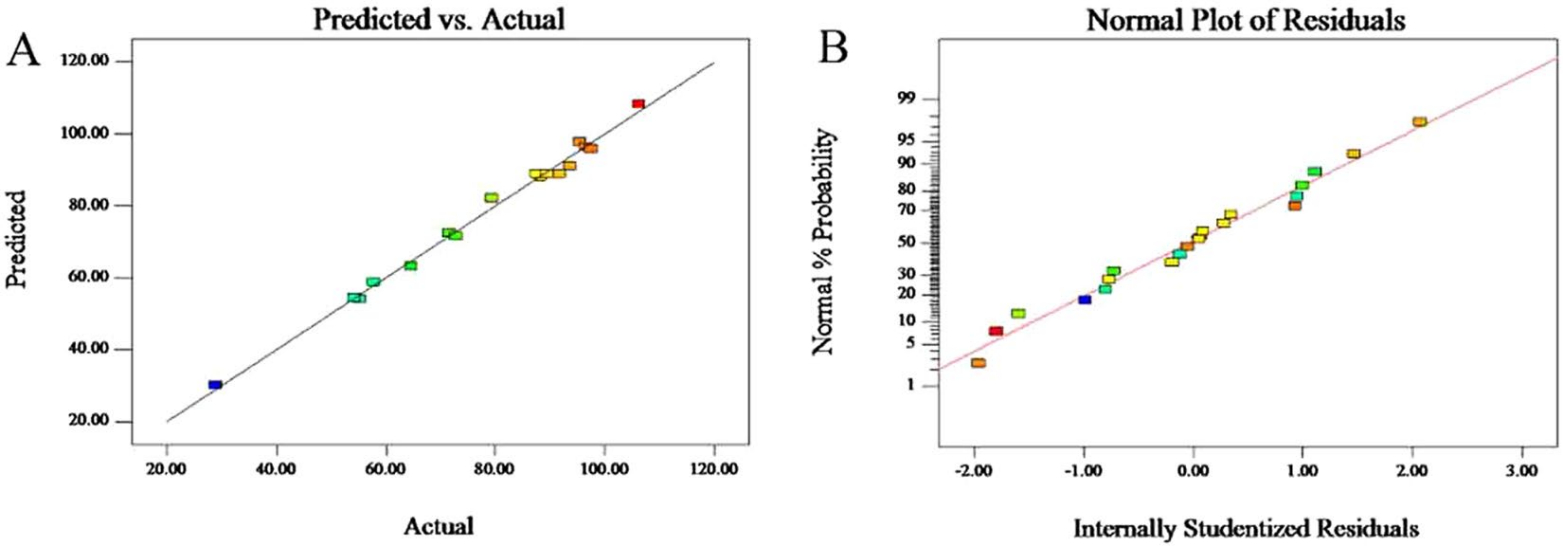
Regression line of predicted vs. actual (**A**) and normal plot of residuals (**B**).

The observed experimental value versus predicted value displays the real responses’ data plotted against the predicted responses (Fig. 4A). The regression line was with high regression coefficients (*R* ^2^ = 0.993). The experimental data points were well distributed close to a straight line, suggesting a relatively excellent relationship between the experimental and predicted values of the responses, and the underlying assumptions of the above analysis are appropriate^39^.

In addition, the adequacy of the models was also evaluated by the residuals. Residuals are thought as elements of variation unexplained by the fitted model and then it is expected that they occur according to a normal distribution. Normal probability plots are a suitable graphical method for judging the normality of the residuals^31, 40^. The observed residuals were plotted against the expected values, given by a normal distribution (Fig. 4B). The approximate straight lines obtained indicated that residuals were normally distributed. Residuals should also present structureless patterns when plotted against predicted values, showing no increase as the size of the fitted value increases. Based on these plots, the residuals appeared to be randomly scattered.

### Response surface and contour plots

The three dimensional response surface plots can provide useful information about the behavior of the system within the experimental design, and facilitate an examination of the effects of the experimental factors on the responses and contour plots between the factors^41^.

**Figure 5:**
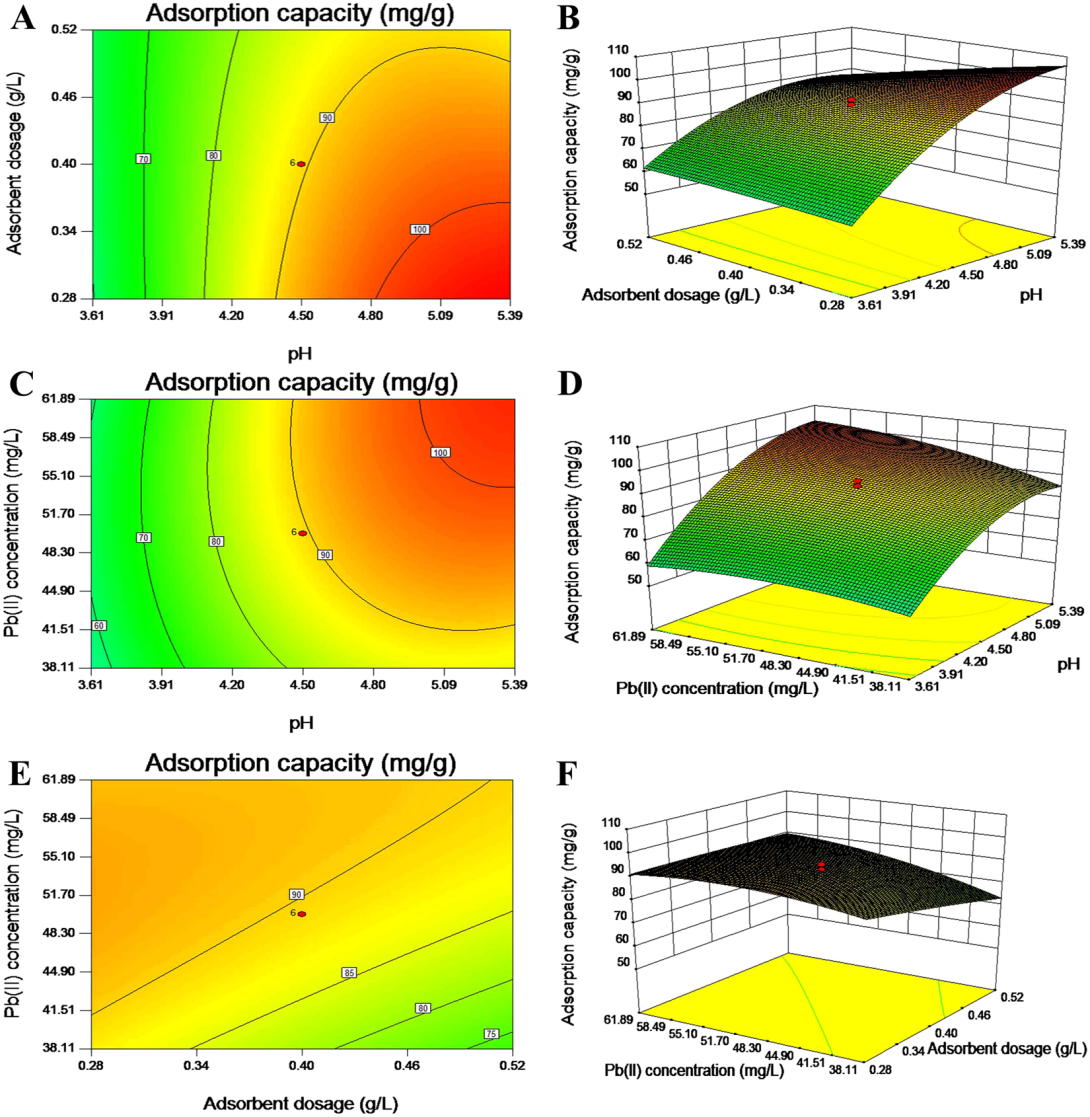
Contour plots and response surface for adsorption capacity (mg/g) in uncoded values for *t* = 3 h. (**A** and **B**) *X*_1_ (pH) and *X*_2_ (adsorbent dosage) in fixed *X*_3_ (Pb(II) concentration) at 50 mg/L; (**C** and **D**) *X*_1_ (pH) and *X*_3_ (Pb(II) concentration) in fixed *X*_2_ (adsorbent dosage) at 0.4 mg/L; (**E** and **F**): X_2_ (adsorbent dosage) and *X*_3_ (Pb(II) concentration) in fixed *X*_1_ (pH) at 4.5.

In Fig. 5A and B, the effect of pH and adsorbent dosage on adsorption capacity was shown at initial Pb(II) concentration of 50 mg/L. It could be observed that the adsorption capacity had no significant change at low initial pH value, and it had a remarkable increase with adsorbent dosage decreasing at higher initial pH value. If kept adsorbent dosage constant, the conclusion could be drawn that the pH higher than 5 was suitable value. The conclusion also could be obtained from the effect of pH and initial Pb(II) concentration on adsorption capacity (shown in Fig. 5C and D). Figure 5E and F represented the effect of adsorbent dosage and initial Pb(II) concentration on adsorption capacity at pH of 4.5. An increase in initial Pb(II) concentration led to increase in the amount of metal adsorption on MCMC-PEI, and the adsorption capacity increased with increasing adsorbent dosage, which corresponded to ordinary rules of adsorption.

### Adsorption kinetics

**Figure 6:**
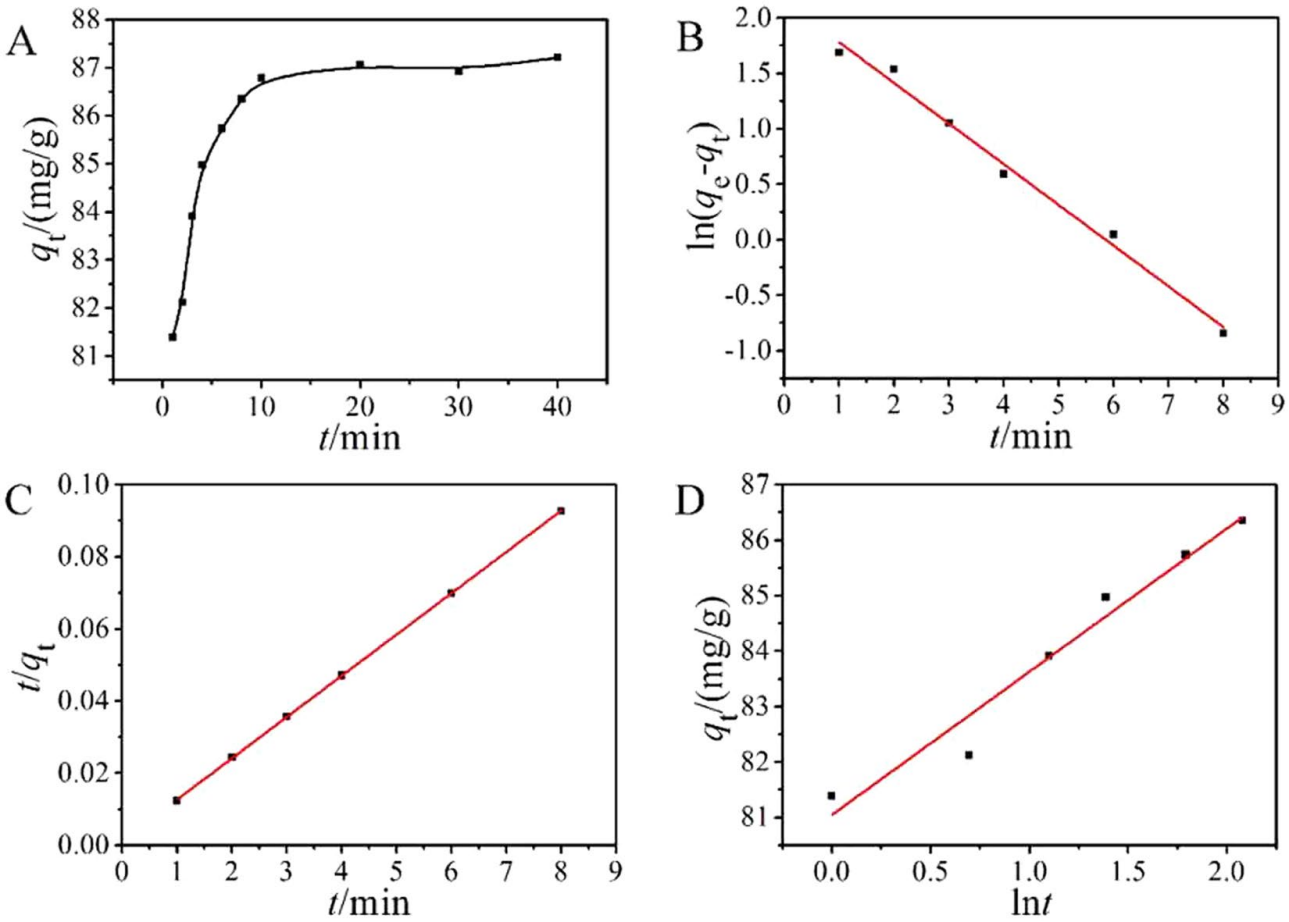
Effects of contact time (**A**), pseudo-first-order kinetics fit (**B**), pseudo-second-order kinetics fit (**C)** and Elovich kinetics fit of Pb(II) adsorption on MCMC-PEI.

Kinetics of the adsorption process is vital in wastewater treatment as it provides essential information on the solute uptake rate and the reaction pathways. The adsorption of Pb(II) on MCMC-PEI as a function of contact time was shown in Fig. 6A. It showed that the removal efficiency of Pb(II) reached equilibrium rapidly at 10 min. It was a fairly short adsorption time. This fast adsorption process of Pb(II) indicated that the surface complexation or chemical reaction was the main mechanism^42^.

In order to investigate the adsorption kinetics in detail, the adsorption kinetics data of Pb(II) were analyzed by testing pseudo-first order kinetic model, pseudo-second order kinetic model and Elovich kinetic model, which can be expressed as follows:

Pseudo-first-order kinetic model:

5 $$\mathrm{ln}({q}_{{\rm{e}}}-{q}_{{\rm{t}}})=\,\mathrm{ln}\,{q}_{{\rm{e}}}-{k}_{{\rm{1}}}t$$

Pseudo-second-order kinetic model:

6 $$\frac{t}{{q}_{t}}=\frac{1}{{k}_{2}{q}_{e}^{2}}+\frac{t}{{q}_{e}}$$

Elovich kinetic model:

7 $$\,{q}_{t}=(\frac{1}{\beta })\mathrm{ln}(\alpha \beta )+(\frac{1}{\beta })\mathrm{ln}\,t$$

Where *q*_t_ (mg/g) and *q*_e_ (mg/g) are the adsorption capacities at time *t* (min) and equilibrium, respectively. *k*_1_ (min^−1^) and *k*_2_ (g/(mg min)) are the pseudo-first-order and pseudo-second-order rate constant, *α* (mg/(g min)) and *β* (g/mg) represent the initial adsorption rate and desorption constant in Elovich model.

The fitted curves were shown in Fig. 6B–D, and the parameters were calculated and listed in Table 2. From the fitting results, we could find that the measured kinetic data of Pb(II) adsorbed by MCMC-PEI fitted pseudo-second order kinetic model with a correlation coefficient of 0.9999. Moreover, the experimental equilibrium adsorption capacity of Pb(II) (86.79 mg/g) fitted well with the calculated value (87.34 mg/g) of pseudo-second-order kinetic model. It suggested that the kinetics of Pb(II) on MCMC-PEI followed the pseudo-second-order model, indicating the chemisorption of the adsorption process.

**Table 2:** Constants and correlation coefficients for the kinetic models.

Model	Parameters	*R* ^2^
Pseudo-first-order	*q*_e_ = 8.564 mg/g	0.9877
	*k*_1_ = 0.3668 min^−1^	
Pseudo-second-order	*q*_e_ = 87.34 mg/g	0.9999
	*k*_2_ = 0.1066 g/(mg min)	
Elovich	*α* = 1.102 × 10^14^ mg/(g min)	0.9529
	*β* = 0.3872 g/mg	

### Adsorption isotherm

The adsorption isotherm of MCMC-PEI for Pb(II) were investigated under the optimal conditions obtained before over a wide range of initial concentration of Pb(II) (40–200 mg/L) at 303 K. In order to understand the adsorption mechanism better, three isotherm equations were selected for the study of modeling these adsorption isotherm data: Langmuir, Freundlich, and Langmuir-Freundlich equations.

Langmuir model assumes that the bulk phases and surfaces of homogeneous sorbents exhibit an ideal behavior with all the adsorption sites identically and energy equivalently. It has been widely used to describe the monolayer and short-term adsorption processes, which is expressed as:

8 $${q}_{e}=\frac{{K}_{L}{q}_{m}{c}_{e}}{1+{K}_{L}{c}_{e}}$$

where *q*_e_ (mg/g) is the amount of Pb(II) adsorbed on MCMC-PEI at equilibrium, *q*_m_ (mg/g) is the saturated adsorption capacity of Pb(II) adsorbed on MCM-PEI to form a complete monolayer coverage, *K*_L_ (L/mg) is the Langmuir adsorption coefficient representing enthalpy of adsorption and varies with temperatures.

Freundlich model is one of the earliest empirical equations used to describe equilibria data, and is very popularly used in the description of adsorption, which is expressed as:

9 $${q}_{e}={K}_{{\rm{F}}}{{c}_{{\rm{e}}}}^{1/n}$$

where *K*_F_ is the Freundlich adsorption coefficient related to the adsorption capacity, *n* is an indicator of isotherm nonlinearity corresponded to the adsorption intensity at specific temperatures. The larger is this value of *n*, the more nonlinear of the adsorption isotherm becomes as its behavior deviates further away from the linear isotherm.

Langmuir-Freundlich model is a combined form of Langmuir and Freundlich expression for the prediction of the heterogeneous adsorption. At low adsorbate concentration, it reduces to Freundlich isotherm, whereas it predicts a monolayer adsorption capacity characteristic of Langmuir isotherm at high adsorbate concentration. It is expressed as follows:

10 $${q}_{{\rm{e}}}=\frac{{q}_{m}{(b{c}_{e})}^{1/{n}^{\text{'}}}}{1+{(b{c}_{e})}^{1/{n}^{\text{'}}}}$$

where *b* (L/mg) is the Langmuir-Freundlich isotherm constant and the parameter *n*′ could be regarded as the parameter characterizing the system heterogeneity. The larger is this parameter *n*′, the more heterogeneous is the system.

**Figure 7:**
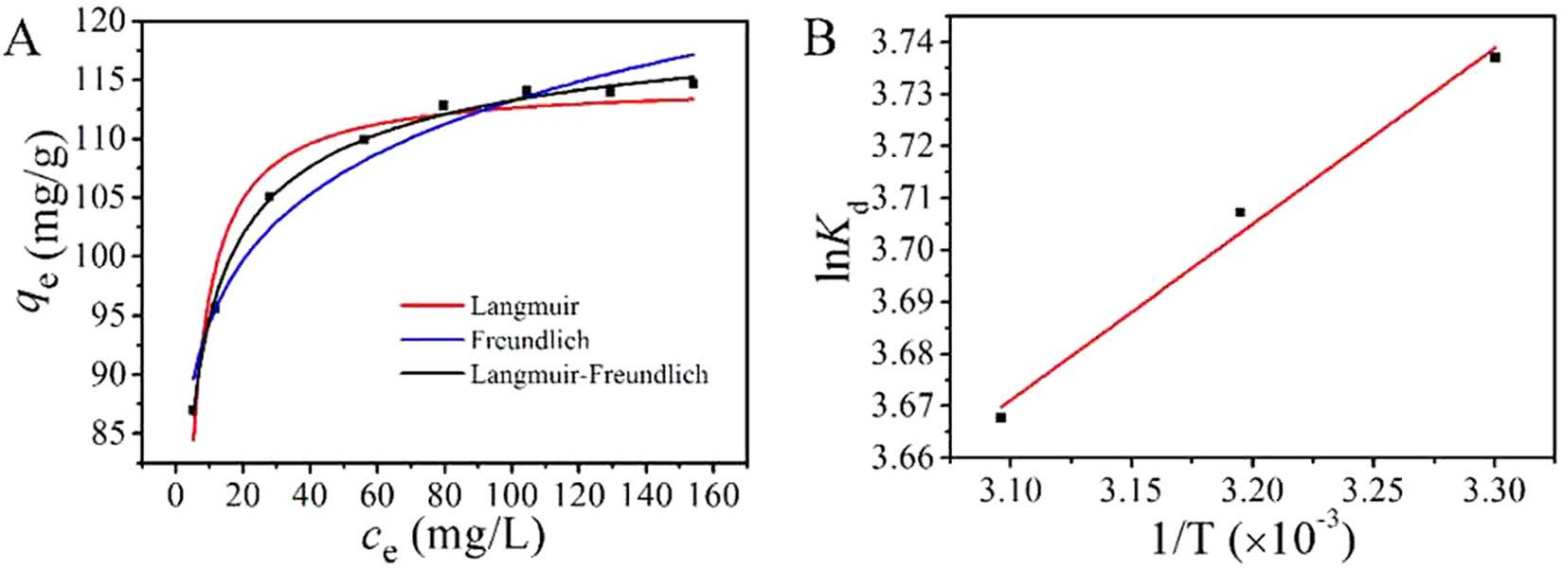
Adsorption isotherms fit (**A**) and adsorption thermodynamics of Pb(II) onto MCMC-PEI.

The fitting results getting from the isotherms were shown in Fig. 7A, and the values of correlation coefficients and other parameters obtained from the adsorbent were given in Table 3. As can be seen from the adsorption isotherms (Fig. 7A) and the correlation coefficients (*R* ^2^) (Table 3), Langmuir-Freundlich model fitted the adsorption isotherm of Pb(II) best among the three isotherm models. According to the curves fitted by the Langmuir-Freundlich model, the *q*_m_ values of Pb(II) adsorbed on MCMC-PEI was 124.0 mg/g, which was relatively high compared with other reported magnetic adsorbents (Table 4).

**Table 3:** Constants and correlation coefficients of adsorption isotherms for the adsorption.

Model	Parameters	*R* ^2^
Langmuir	*q*_m_ = 114.7 mg/g	0.9531
	*K*_L_ = 0.5338 L/mg	
Freundlich	*K*_F_ = 78.61	0.9534
	*n* = 12.62	
Langmuir-Freundlich	*q*_m_ = 124.0 mg/g	0.9952
	*b* = 0.9831 L/mg	
	*n*′ = 1.952

**Table 4:** Maximum adsorption capacity for the adsorption of Pb(II) onto various magnetic adsorbents reported recently.

Adsorbent	Adsorption capacity (mg/g)	Reference
Magnetic diatomite-CNTs	60.8	43
MLP	78.74	44
ZnO@SiO_2_@Fe_3_O_4_/C	94.3	45
MNP–CTS	140.4	46
Co_0.6_Fe_2.4_O_4_ nanoparticles	80.32	47
Fe_3_O_4_@SiO_2_-IIP	32.58	48
N-Fe/OMC	159.93	49
MCMC-PEI	124.0	This work

### Adsorption thermodynamics

The thermodynamic parameters for the adsorption of Pb(II) onto MCMC-PEI can be calculated from the temperature-dependent adsorption isotherms, which were carried out at temperatures ranging from 303 K to 323 K. The Δ*G* was calculated with Eq. (11). Δ*H* and Δ*S* were calculated from the slope and intercept of the plot of ln *K*_*d*_ versus 1/*T* using Eq. (12).

11 $${\rm{\Delta }}G=-RT\,\mathrm{ln}\,{K}_{d}$$

12 $$\mathrm{ln}\,{K}_{d}=\frac{{\rm{\Delta }}S}{R}-\frac{{\rm{\Delta }}H}{RT}$$

Where Δ*G* (kJ/mol) is the Gibbs free energy change of adsorption, Δ*H* (kJ/mol) is the enthalpy change, Δ*S* (J/mol K) is the entropy change, *T* (K) is the temperature in Kelvin, *R* (8.314 J/mol K) is universal gas constant, and *K*_d_ (L/g) is the thermodynamic equilibrium constant which was computed by plotting ln(*q*_e_/*c*_e_) versus *q*_e_ and extrapolating *q*_e_ to zero.

The linear plot of ln *K*_d_ versus 1/*T* and thermodynamic parameters were listed in Fig. 7B and Table 5. The negative value of Δ*G* and Δ*H* indicated that the adsorption of Pb(II) onto MCMC-PEI was a spontaneous and exothermic process. It revealed that the exothermic adsorption of Pb(II) onto MCMC-PEI was subdued by an increase in temperature. The positive value of Δ*S* stated clearly that the randomness increased at the solid-solution interface during the Pb(II) adsorption. During the adsorption process, the coordinated water molecules that were displaced by the Pb(II) species gained more translational entropy than the lost by Pb(II) species, resulting in increased randomness in the metal ions-adsorbent interaction^50^.

**Table 5:** Thermodynamic parameters of the adsorption of Pb(II) by MCMC-PEI.

*T* (K)	Δ*G* (kJ/mol)	Δ*H* (kJ/mol)	Δ*S* (J/(mol·K))
303	−9.414	−2.813	21.80
313	−9.647		
323	−9.850		

### Performance evaluation

Considering the coexisting metal ions such as K^+^, Na^+^, Ca^2+^ and Mg^2+^ in the actual water body, the effect of them for adsorption efficiency was investigated. Solutions containing K^+^, Na^+^, Ca^2+^ and Mg^2+^ were prepared by adding potassium nitrate, sodium nitrate, calcium nitrate and magnesium nitrate into lead ions solution, respectively. The results indicated that the coexisting K^+^, Na^+^, Ca^2+^ and Mg^2+^ had no evident influence on Pb(II) adsorption.

**Figure 8:**
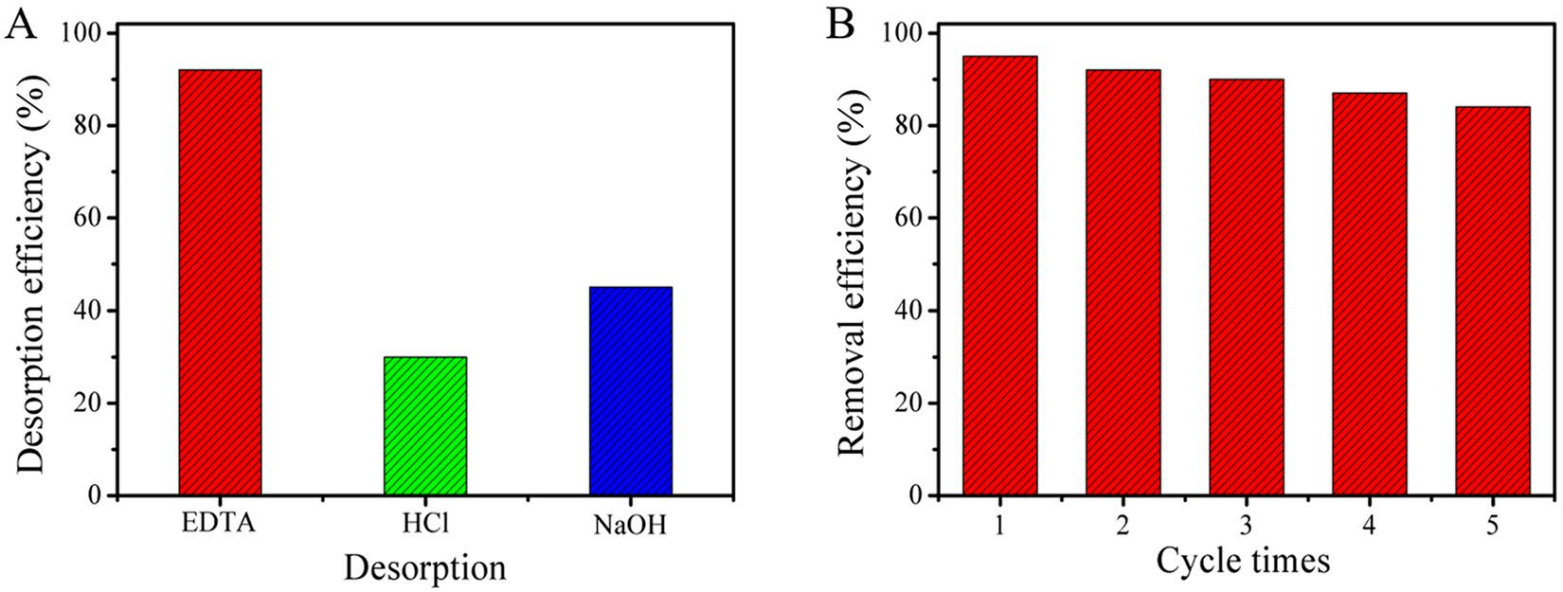
Different desorbents to release Pb(II) from MCMC-PEI (**A**) and the reusability of MCMC-PEI for five cycles by using EDTA (**B**).

In addition, the recycling and regeneration abilities of the adsorbent are important for evaluating their performance for practical applications. Herein, EDTA (0.1 mol/L), HCl (0.1 mol/L) and NaOH (0.1 mol/L) were used to desorb the adsorbed Pb(II). As a result, EDTA was observed to be an effective desorbent to recover Pb(II) from MCMC-PEI (Fig. 8A), which was applied for the succeeding regeneration experiment. Meanwhile, the adsorption-desorption experiments were carried out with the MCMC-PEI dosage of 0.6 g/L and the initial Pb(II) concentration of 50 mg/L at 298 K and pH 4.5. Observed from the Fig. 8B, the MCMC-PEI could still remain about 85% of its initial adsorption capacity after five adsorption-desorption recycle experiments. The decrease of adsorption capacity of MCMC-PEI might be ascribed to the loss of the adsorbent or the irreversible occupation of part adsorption sites. The good reusability suggested that the prepared MCMC-PEI adsorbent could be potentially applied for practical wastewater treatment.

## Conclusion

In the present study, novel MCMC-PEI adsorbent was obtained and utilized for the removal of Pb(II) in aqueous solution. The results revealed that the adsorption process depended on the pH of solution strongly. Meanwhile, the adsorption kinetics fitting results showed that the adsorption process was in accordance with the pseudo-second-order kinetic model well, suggesting that the adsorption was mainly determined by the chemical adsorption process. Adsorption isotherm fitting results manifested that the adsorption process could be well described by Langmuir-Freundlich model, and the adsorption capacity reached 124.0 mg/g which was better than some other reports. Furthermore, thermodynamic studies illustrated that the adsorption of Pb(II) was a spontaneous and exothermic process. Simultaneously, good reusability of the adsorbent was obtained by the adsorption-desorption experiment. Therefore, the prepared MCMC-PEI adsorbent could be a potential candidate in the treatment of Pb(II) wastewater.

## Electronic supplementary material

Supplementary materials
