# Supplementary material for: Rapid removal of Pb(II) from aqueous solution using branched polyethylenimine enhanced magnetic carboxymethyl chitosan optimized with response surface methodology
Source: Sci Rep. 2017 Aug 31;7:10264. doi: 10.1038/s41598-017-09700-5 (PMC5579235; doi:10.1038/s41598-017-09700-5)
Supplement: Supplementary file 1 — Supplementary materials [file 41598_2017_9700_MOESM1_ESM.doc]

***Supplementary materials***

**Rapid removal of Pb(II) from aqueous solution using branched** **polyethylenimine enhanced magnetic carboxymethyl chitosan optimized with** **response surface methodology**

Yaoguang Wang^a^, Di Wu^b^, Qin Wei^a^, Dong Wei^b^, Tao Yan^b^, Liangguo Yan^b^, Lihua Hu^a*^, Bin Du^a^

^a^ Key Laboratory of Interfacial Reaction & Sensing Analysis in Universities of Shandong, School of Chemistry and Chemical Engineering, University of Jinan, Jinan 250022, China

^b^ School of Resources and Environment, University of Jinan, Jinan 250022, China

^*^Corresponding author. Tel. + 86 531 82767872; fax: + 86 531 82767367.

E-mail address: hulihua1206@163.com

**Table S1** The 3-factor central composite design matrix and values of response

| Run | *X*_1_, pH | *X*_2_, Adsorbent dosage (g/L) | *X*_3_, Pb(II) concentration (mg/L) | *Y*, Adsorption capacity (mg/g) | |
| --- | --- | --- | --- | --- | --- |
|  |  |  |  | Experimental | Predicted |
| 1 | 4.50 | 0.20 | 50.00 | 97.49 | 95.91 |
| 2 | 4.50 | 0.40 | 50.00 | 87.52 | 89.00 |
| 3 | 4.50 | 0.40 | 50.00 | 89.54 | 89.00 |
| 4 | 3.61 | 0.52 | 61.89 | 64.61 | 63.27 |
| 5 | 4.50 | 0.60 | 50.00 | 79.39 | 82.08 |
| 6 | 5.39 | 0.52 | 38.11 | 71.57 | 72.44 |
| 7 | 3.61 | 0.52 | 38.11 | 55.25 | 54.11 |
| 8 | 5.39 | 0.28 | 38.11 | 95.42 | 97.77 |
| 9 | 5.39 | 0.52 | 61.89 | 96.50 | 96.55 |
| 10 | 5.39 | 0.28 | 61.89 | 106.22 | 108.37 |
| 11 | 4.50 | 0.40 | 50.00 | 91.83 | 89.00 |
| 12 | 6.00 | 0.40 | 50.00 | 93.71 | 90.97 |
| 13 | 4.50 | 0.40 | 50.00 | 89.66 | 89.00 |
| 14 | 3.61 | 0.28 | 61.89 | 54.24 | 54.39 |
| 15 | 4.50 | 0.40 | 30.00 | 72.84 | 71.53 |
| 16 | 3.61 | 0.28 | 38.11 | 57.79 | 58.75 |
| 17 | 4.50 | 0.40 | 50.00 | 88.63 | 89.00 |
| 18 | 4.50 | 0.40 | 70.00 | 88.26 | 88.14 |
| 19 | 3.00 | 0.40 | 50.00 | 28.87 | 30.17 |
| 20 | 4.50 | 0.40 | 50.00 | 89.10 | 89.00 |

**Table S2** ANOVA of the quadratic model before amendment

| Source | Sum of Squares | df | Mean  aquare | *F* value | *p* value Prob>F |
| --- | --- | --- | --- | --- | --- |
| Model | 6997.95 | 9 | 777.55 | 180.49 | < 0.0001 |
| *x*_1_-pH | 4462.84 | 1 | 4462.84 | 1035.92 | < 0.0001 |
| *x*_2_-Adsorbent dosage | 231.09 | 1 | 231.09 | 53.64 | < 0.0001 |
| *x*_3_-Pb(II) concentration | 333.24 | 1 | 333.24 | 77.35 | < 0.0001 |
| *x*_1_*x*_2_ | 214.34 | 1 | 214.34 | 49.75 | < 0.0001 |
| *x*_1_*x*_3_ | 111.84 | 1 | 111.84 | 25.96 | 0.0005 |
| *x*_2_*x*_3_ | 91.32 | 1 | 91.32 | 21.20 | 0.0010 |
| *x*_1_^2^ | 1469.81 | 1 | 1469.81 | 341.17 | < 0.0001 |
| *x*_2_^2^ | 3.61 | 1 | 3.61 | 0.84 | 0.3818 |
| *x*_3_^2^ | 155.92 | 1 | 155.92 | 36.19 | 0.0001 |
| Residual | 43.08 | 10 | 4.31 |  |  |
| Lack of Fit | 32.86 | 5 | 6.57 | 3.21 | 0.1129 |
| Pure Error | 10.22 | 5 | 2.04 |  |  |
| Cor Total | 7041.04 | 19 |  |  |  |
| *R*^2^= 0.9939, Adj *R*^2^= 0.9884, Pred *R*^2^= 0.9624, Adeq Precision=52.97 | | | | | |

The significance of the coefficient term is determined by the value of *F* and *p*, and the larger the value of *F* and the smaller the value of *p*, the more significant is the corresponding coefficients ^1^. The *F* value of Fischer was obtained by the relationship between the variance due to the regression and the residual variance (*F* value = S^2^_reg_/S^2^_err_). If the model was a good predictor of the experimental results, *F* value should be greater than the tabulated value of the *F*-distribution for a certain number of degrees of freedom in the model at a level of significance α ^2^. *F*-ratios obtained for adsorption capacity (180.49) were clearly greater than the value of tabular *F* value (*F*_0.05(9,10) tabular_ = 3.02) at the 5% level, indicating that the treatment differences are highly significant. Prob>F is the probability that all the variation in the results were due to random error ^3^, and thus the very low probability values (<0.0001) obtained for both two responses indicate that results were not random and the models is significant. The *p* is lower than 0.05, suggesting the model was considered to be statistically significant ^4^.

**Table S3** ANOVA of the quadratic model after amendment

| Source | Sum of Squares | df | Mean  aquare | *F* value | *p* value Prob>F |
| --- | --- | --- | --- | --- | --- |
| Model | 6994.35 | 8 | 874.29 | 205.99 | < 0.0001 |
| *x*_1_-pH | 4462.84 | 1 | 4462.84 | 1051.50 | < 0.0001 |
| *x*_2_-Adsorbent dosage | 231.09 | 1 | 231.09 | 54.45 | < 0.0001 |
| *x*_3_-Pb(II) concentration | 333.24 | 1 | 333.24 | 78.52 | < 0.0001 |
| *x*_1_*x*_2_ | 214.34 | 1 | 214.34 | 50.50 | < 0.0001 |
| *x*_1_*x*_3_ | 111.84 | 1 | 111.84 | 26.35 | 0.0003 |
| *x*_2_*x*_3_ | 91.32 | 1 | 91.32 | 21.52 | 0.0007 |
| *x*_1_^2^ | 1469.88 | 1 | 1469.88 | 346.32 | < 0.0001 |
| *x*_3_^2^ | 152.75 | 1 | 152.75 | 35.99 | < 0.0001 |
| Residual | 46.69 | 11 | 4.24 |  |  |
| Lack of Fit | 36.46 | 6 | 6.08 | 2.97 | 0.1261 |
| Pure Error | 10.22 | 5 | 2.04 |  |  |
| Cor Total | 7041.04 | 19 |  |  |  |
| *R*^2^= 0.9934, Adj *R*^2^= 0.9885, Pred *R*^2^= 0.9675, Adeq Precision=56.59 | | | | | |

The correlation coefficient (*R*^2^) quantitatively evaluates the correlation between the experimental data and the predicted responses. In this study, the values of the correlation coefficient *R*^2^=0.9934, indicating that 99.34% of the variability in the response could be explained by the regression models. The adjusted correlation coefficient (adjusted *R*^2^) was a measure of goodness of a fit, but it corrected the *R*^2^ for the sample size and the number of terms in the model by using the degrees of freedom on its computations. If there were many terms in the model and the sample size was not very large, the adjusted *R*^2^ may be noticeably smaller than the *R*^2^ value ^2^. Here, the adjusted *R*^2^ values (0.9885) were also very high to advocate for a high significance of the models, which ensured a satisfactory adjustment to the polynomial model to the experimental data. “Adequacy precision” measured the signal-to-noise ratio. It is reported that a ratio greater than 4 is desirable ^5^. The ratio of 56.59 indicated an adequate signal. This model could be used to navigate the design space.

**References:**

1. Amini, M. et al. Application of response surface methodology for optimization of lead biosorption in an aqueous solution by Aspergillus niger. *J. Hazard. Mater.* **154**, 694-702 (2008).

2. Santos, S.C. & Boaventura, R.A. Adsorption modelling of textile dyes by sepiolite. *Appl. Clay Sci.* **42**, 137-145 (2008).

3. Fathinia, M., Khataee, A., Zarei, M. & Aber, S. Comparative photocatalytic degradation of two dyes on immobilized TiO_2_ nanoparticles: effect of dye molecular structure and response surface approach. *J. Mol. Catal. A: Chem.* **333**, 73-84 (2010).

4. Kim, H., Kim, J., Cho, J. & Hong, J. Optimization and characterization of UV-curable adhesives for optical communications by response surface methodology. *Polym. Test.* **22**, 899-906 (2003).

5. Muthukumar, M., Mohan, D. & Rajendran, M. Optimization of mix proportions of mineral aggregates using Box Behnken design of experiments. *Cem. Concr. Compos.* **25**, 751-758 (2003).
